# Human Embryonic Stem Cell-Derived Oligodendrocyte Progenitors Aid in Functional Recovery of Sensory Pathways following Contusive Spinal Cord Injury

**DOI:** 10.1371/journal.pone.0047645

**Published:** 2012-10-16

**Authors:** Angelo H. All, Faith A. Bazley, Siddharth Gupta, Nikta Pashai, Charles Hu, Amir Pourmorteza, Candace Kerr

**Affiliations:** 1 Department of Biomedical Engineering, Johns Hopkins University School of Medicine, Baltimore, Maryland, United States of America; 2 Department of Neurology, Johns Hopkins University School of Medicine, Baltimore, Maryland, United States of America; 3 Department of Biochemistry and Molecular Biology, University of Maryland, Baltimore, Maryland, United States of America; Universidade Federal do Rio de Janeiro, Brazil

## Abstract

**Background:**

Transplantations of human stem cell derivatives have been widely investigated in rodent models for the potential restoration of function of neural pathways after spinal cord injury (SCI). Studies have already demonstrated cells survival following transplantation in SCI. We sought to evaluate survival and potential therapeutic effects of transplanted human embryonic stem (hES) cell-derived oligodendrocyte progenitor cells (OPCs) in a contusive injury in rats. Bioluminescence imaging was utilized to verify survivability of cells up to 4 weeks, and somatosensory evoked potential (SSEPs) were recorded at the cortex to monitor function of sensory pathways throughout the 6-week recovery period.

**Principal Findings:**

hES cells were transduced with the firefly luciferase gene and differentiated into OPCs. OPCs were transplanted into the lesion epicenter of rat spinal cords 2 hours after inducing a moderate contusive SCI. The hES-treatment group showed improved SSEPs, including increased amplitude and decreased latencies, compared to the control group. The bioluminescence of transplanted OPCs decreased by 97% in the injured spinal cord compared to only 80% when injected into an uninjured spinal cord. Bioluminescence increased in both experimental groups such that by week 3, no statistical difference was detected, signifying that the cells survived and proliferated independent of injury. Post-mortem histology of the spinal cords showed integration of human cells expressing mature oligodendrocyte markers and myelin basic protein without the expression of markers for astrocytes (GFAP) or pluripotent cells (OCT4).

**Conclusions:**

hES-derived OPCs transplanted 2 hours after contusive SCI survive and differentiate into OLs that produce MBP. Treated rats demonstrated functional improvements in SSEP amplitudes and latencies compared to controls as early as 1 week post-injury. Finally, the hostile injury microenvironment at 2 hours post-injury initially caused increased cell death but did not affect the long-term cell proliferation or survival, indicating that cells can be transplanted sooner than conventionally accepted.

## Introduction

Spinal cord injury (**SCI**) results in neuronal degeneration and demyelination due to oligodendrocyte apoptosis at the region of trauma and causes severe functional impairment of motor and sensory pathways. Cell replacement therapy offers an avenue for the restoration of function by replacing lost oligodendrocytes. A number of recent studies have focused on regeneration of damaged axons and lost neural cells to potentially treat SCI using a variety of stem cell-derived neural cell types [1]–[4]. These studies have suggested that stem cells could potentially improve locomotor function after SCI following transplantations of human neural stem cells (**NSCs**) [5], gliogenic secondary neurospheres [6], and motor neuron progenitors [7]. The impetus of such work resulted in the first FDA approved clinical trial using human embryonic stem (**hES**) cells by Geron in 2010 [8], although the trial was halted due to economic considerations on November 11, 2011.

To optimize cell-based treatment, current remyelination strategies are focusing on the behavior of transplanted cells *in vivo*. We have previously demonstrated the ability to isolate and differentiate oligodendrocyte progenitor cells (**OPCs**) from hES cells with high purity [9] and have shown that they can survive and differentiate into oligodendrocytes (**OLs**) when injected into the spinal cord hours after acute SCI [10]. Several other studies have shown that human-derived OPCs injected weeks or months following SCI induce motor behavior improvements [11]–[14]. In these studies, remyelination and functional recovery was assessed using motor behavioral tests. Although these assessments reveal gross locomotor recovery, they are not sensitive to small improvements in neural conductivity or remyelination of sensory pathways in the spinal cord following trauma.

An issue raised by several SCI cell therapy studies is the survivability of the grafted cells after transplant. This analysis is critical as the time after injury at which cells are injected is currently a topic of debate. Most of the work in the SCI field has assumed that cells will not survive a hostile environment immediately after injury and these studies have suggested that delayed transplantation, about 7 to 9 days after injury, leads to better cell survival and migration than immediate transplantations [15]. Moreover, transplantations with mesenchymal stem cell derivatives suggest that these cells do not survive long after transplant [16]. However, very little is known regarding the behavior of OPCs after transplantation or their reaction to the injured environment. Therefore, cell tracking studies are critical in determining the optimal number of cells to be injected, as well as determining the most appropriate time post-injury to inject the cells.

Electrophysiological assessments provide objective tools for monitoring recovery after SCI in both human subjects and animal models. These assessments target either the motor or sensory pathways. For instance, recovery of motor evoked potentials (**MEP**) measures the function of descending motor pathways. Improvements in MEP have been shown in rodent studies of SCI after human stem cell-derived transplantation [11], [17]. However, studies have not been performed using somatosensory evoked potentials (**SSEP**), which assess the function of ascending pathways from the periphery to the primary somatosensory cortex (**S1**). This is important as the majority of SCIs in humans are contusion injuries, which predominantly damage the dorsal regions of the spinal cord that consist primarily of the ascending sensory pathways. Therefore, in incomplete SCIs in humans, the dorsal sensory pathways undergo significant damage. In rats, these regions also consist of primary ascending pathways and are the focal point of damage in rat SCI contusive models. Thus, rats provide a robust model for studying the impact of contusive injury in evaluating SCI injury progression and recovery.

SSEP evaluations are sensitive measures of recovery as slight increases in sensory conduction can lead to significant improvements in sensory function and thus the quality of life of patients with SCI. For this reason, SSEPs are used in the operating room by clinicians to monitor surgical procedures and to assess functional outcomes of recovery [18], [19]. Therefore, the experimental outcomes of SSEP are highly translatable to the clinic and to patients with SCI and here, we utilized SSEP measures to determine whether injected human ES derived OPCs into these damaged ascending sensory pathways improve their conduction.

This approach was combined with *in vivo* bioluminescence imaging (**BLI**) using a firefly luciferase reporter [20], [21] in order to track transplanted cells. BLI is beneficial over other *in vivo* imaging techniques due to its simplicity and high sensitivity for detecting survived cells [22]. Moreover, we used a lentiviral integration of the luciferase gene into the cellular genome, so that it can be continually produced. This allowed for long term *in vivo* monitoring to measure survival and migration without the issue of other cell labeling methods in which the label diffuses out over time.

The purpose of this study was to determine whether transplantation of hES cell-derived OPCs can aid in the repair of sensory tracts after contusive SCI in rats. We also aimed to track the grafted cells non-invasively and evaluate whether microenvironment at 2 hours post-injury significantly affected cell survival. We showed that the cells were detectable 4 weeks after transplantation in both injured and non-injured groups. Electrophysiological assessments up to 6 weeks demonstrated for the first time that the grafted cells may aid in reducing the immediate secondary injury or spinal shock within the first week and promote repair of sensory pathways. Histological analyses verified the differentiation of OPCs into MBP-producing OLs. This is the first report to show the live progression of OPC survival after injection into a hostile environment of SCI and correlate their survival with SSEP improvements, a proven *in vivo* method of measuring somatosensory recovery. Two phases of SSEP recovery were detected; the early SSEP recovery is consistent with reduced inflammation, while long-term SSEP recovery may be associated with the remyelination detected via histological examinations. Thus, increases in SSEP also corresponded with improved tissue integrity and myelin staining in the hES-OPC treated rats.

## Methods

### Animals

All procedures were performed in accordance with protocols approved by the Institutional Animal Care and Use Committee of the Johns Hopkins University. A total of 33 adult female Lewis rats (200–230 g, aged 6–8 weeks) were used in this study. Rats were housed in individual cages and provided food and water *ad libitum* throughout the study.

### Cell Culture

H1 (WA01) cells from WiCell (Madison, WI) were expanded in ES cell growth media and differentiated according to our laboratory’s established protocols [9], [10]. Embryoid bodies (**EBs**) were formed by suspending undifferentiated ES cell colonies in ES growth media on ultra low adhesion culture dishes. The neural differentiation was commenced by growing EBs in N2B27 medium [23], supplemented with 200 ng/ml noggin, 20 ng/ml FGF2, and 20 ng/ml FGF4 (all from R&D Systems). EBs were grown for 15 days and then plated on matrigel and grown in N2B27 media supplemented with 20 ng/ml FGF2 for 5 days, resulting in the appearance of neural progenitors (**NPs**). NPs were then passaged with 0.05% trypsin/EDTA and cultured for 3 weeks in N2B27 media with 20 ng/ml of PDGF-AA (PeproTech) and 20 ng/ml EGF (R&D Systems) until transplantation. A human fibroblast line (HFF1, from ATCC) from neonate foreskins were cultured in 10% fetal bovine serum as previously described [24], [25].

### Lentiviral Transduction

For constitutive firefly *luciferase* expression, 2.5×10^6^ undifferentiated hES cells (H1 cells from Wicell, Madison WI) were transfected for 6 hours with the lentiviral vector, pLenti4-CMV-fLuc2, with a multiplicity of infection of 10, in the presence of 0.1% polybrene (Chemicon, MA). Cells were cultured on 75 cm^2^ polystyrene tissue culture dishes (Corning, NY) at 37°C and 5% CO_2_.

### Spinal Cord Injury and Post-surgical Care

Our SCI model has been previously described [26]–[34]. Prior to surgery, each rat was anesthetized by intraperitoneal (**i.p.**) injection of a mixture of ketamine (30.43 mg/kg), xylazine (4.35 mg/kg), and acepromazine maleate (0.87 mg/kg). An adequate level of anesthesia was determined by monitoring the corneal reflex and limb withdrawal to pinch stimuli. A laminectomy was performed by removing the thoracic vertebra T7, T8, and T9 to expose the dorsal surface of the spinal cord. Stabilization clamps served to immobilize the T6 and T10 vertebra to support the spinal column during impact. A MASCIS impactor was used to induce the contusion injury. The spinal cord position at T8 was placed directly under the vertical shaft of the mechanical impactor. The height of the impactor (tip diameter: 2 mm, weight: 10 g) was adjusted to 6.25 mm or 12.5 mm. The probe was released and allowed to fall freely onto the spinal cord and cause impact. The impact trajectory, distance, velocity, and time were recorded by a personal computer. Animals for which the error in any of these parameters was greater than 5% were excluded from the study.

Immediately following surgery, rats were given subcutaneous (s.c.) injections of isotonic saline (20 ml/kg), which was administered daily following surgery for 7 days, and they were maintained on an isothermic pad until they were alert and mobile. Rats generally regained consciousness within 30 minutes of the surgical procedure. The rats’ bladders were expressed twice daily, and the rats were inspected for weight loss, discomfort, dehydration, and autophagia, with appropriate veterinary care as needed. Liquid Tylenol and ampicillin were administered for 7 days. All procedures were approved by NIH Guide for the Care and Use of Laboratory Animals, the Guidelines for the Use of Animals in Neuroscience Research and the Johns Hopkins University IACUC.

### Cell Transplantation

To avoid immunological rejection, each rat was immunosuppressed with daily subcutaneous injections of 14 mg/kg cyclosporine A (250 mg/5 ml, Bedford Labs, Bedford, OH) beginning three days prior to OPC transplantation. Approximately 125,000 cells per µL were transplanted two hours after injury using a Hamilton 10 µL syringe (7635-01, Hamilton, Reno, NV) and electronic microsyringe pump (Micro4, World Precision Instruments, Sarasota FL). The cells were injected at a controlled rate of 1 µL/min at three injection sites, 1.5 mm below the surface of the cord. Four microliters of cell suspension (approximately 500,000 cells) was injected at the epicenter of the injury (into the grey matter posterior to the central canal), 2 µL (∼250,000 cells) was injected 4 mm above and 1 mm to the left of the epicenter, and 2 µL (∼250,000 cells) was injected 4 mm below and 1 mm to the right of the epicenter (N = 9). After each injection, the cells were allowed to settle for at least 2 minutes per µL injected before the needle was retracted. The muscle and skin were then sutured and closed in layers, and the rat was given appropriate post-operative treatment. Controls included an injection of phosphate buffer saline only (N = 17), heat-killed OPCs (N = 4), or human neonatal fibroblast injections (N = 3).

### Electrophysiological Assessments

Prior to the start of the experiments, electrode pedestals were implanted into the skull of the 26 rats for electrophysiological assessments, as previously described [27], [29]. Briefly, a standard dental drill was used to drill four burr holes into cranium at locations of the primary somatosensory cortex corresponding to the hindlimbs (2.8 mm lateral, 2.5 mm posterior to bregma) and forelimbs (3.8 mm lateral, 0.2 mm posterior to bregma). A fifth electrode on the right frontal bone was inserted as the intracranial reference. Transcranial screw electrodes (E363/20, Plastics One, Inc., Roanoke, VA) were then screwed into the holes such that they made very light contact with the dura mater, and they were mounted to an electrode pedestal (MS363, Plastics One Inc., Roanoke, VA) using dental cement. A MASCIS Impactor (Rutgers University, NJ) was used to induce 12.5 mm contusion injuries in all 26 rats. Following the contusion, rats received either hES-derived OPC transplants 2 hours after injury (N = 9) or saline only (N = 17). For SSEP recordings, the rats were anesthetized with 1.5% isoflurane. Intramuscular needle electrodes (Safelead F-E3-48, Grass Technologies, West Warwick, RI) were used to electrically stimulate the median and tibial nerves of the forelimbs and hindlimbs, respectively. For each session, each of the 4 limbs was stimulated in an alternating fashion and the corresponding SSEPs were recorded. Stimuli were provided at 1 Hz such that each limb received a pulse at a frequency of 0.25 Hz (3 mA, 200 µs pulse width). A reference ground electrode was placed subdermally at the back of the neck. Signals were amplified with a gain of 20,000 and sampled at 4882 Hz using a custom designed TDT System (Tucker-Davis Technologies, Alachua, FL).

For each recording session, at least 200 sweeps were recorded. Two pre-injury baseline recordings were taken prior to injury for each rat. Following injury, SSEPs were recorded in twenty-minute sessions on days 4, 7, 14, 28, 35, and 42 after injury. Signal processing was performed using MATLAB 7.0 (MathWorks Inc., Natick, MA). To improve signal-to-noise ratio, each sweep was high-pass filtered (20 Hz cutoff), notch filtered (50–70 Hz), and mean corrected. The mean of the first 200 sweeps of each recording session was taken for further analysis, for which the peak-to-peak amplitude and latency to the first positive peak were identified.

### Light Microscopy

Morphological changes were also assessed postmortem in rats which received 12.5 mm contusion injuries by light and transmission electron microscopy (TEM) examinations. These rats received an injection of hES-OPCs or as controls, injected with either heat-killed OPCs or human neonatal fibroblasts. In all cases, animals were deeply anesthetized using isoflurane and perfused via the aorta with a 4% paraformaldehyde (**PFA**)/PBS solution. The spinal cord was extracted from the spinal column and submerged in 4% PFA for 24 **hours**. For light microscopy, fixed spinal cords were embedded in paraffin blocks for (a) hematoxylin and eosin (**H&E**) staining, and (b) Luxol fast blue (**LFB**)/cresyl violet staining, or transferred to a 30% sucrose solution and embedded in optimal cutting temperature (**OCT**) compound (Tissue-Tek, Sakura Finetek, Torrance, CA) for cryostat processing and immunofluorescence staining. LFB is a routine stain for myelin, cresyl violet stains the Nissl bodies of neurons and H&E stains were used to assess cellular density of the damaged cords as performed previously [35], [36].

Immunofluorescence stains were used to identify cell origin, differentiation, and tumorigenic potential. These stains were performed on spinal cords prepared in OCT freezing compound as previously described [10]. Briefly, 10 micron frozen sections were postfixed with 4% PFA for 10 minutes and stained with 1∶50 dilutions of the following antibodies: human specific nuclear stain (**HNA**), myelin basic protein (**MBP**), glial fibrillary acidic protein (**GFAP**), and POU domain, class 5, transcription factor 1 (**POU5F1**) also known as OCT4. All antibodies were purchased from R&D systems except OCT4 which was purchased from BDBiosciences. Primary antibodies were detected using Alexa secondary antibody probes (Molecular Probes) at 1∶200 dilutions as previously described [10]. Spinal cords were roughly 2.5–3 mm in thickness from which approximately 80–100 cuts surrounding the midline of the cords were generated. These sections represented regions above, below and at the center of cell injections for immunological staining. Staining for each antibody was performed in at least triplicate for each rat and at least three rats per treatment were studied for quantitative analysis.

### Transmission Electron Microscopy

Fixed spinal cords were dissected such that the ventral portion of the cord and 0.5 mm on either side of the epicenter of injury were removed and discarded. For transmission electron microscopy, 2 millimeter samples of the remaining portion containing the epicenter and injected sites were post-fixed in 0.8% potassium ferrocyanide that was reduced with 1% osmium tetroxide, 0.1 M sodium cacodylate, and 3 mM CaCl_2_, as previously described [35]. Samples were then embedded in Eponate 12 for sectioning. Spinal cord sections (70 nm width) corresponding to the site of the lesion were cut using a Riechert Ultracut E with a Diatome diamond knife, collected on formvar-coated 1×2 mm^2^ copper grids, and stained with uranyl acetate and lead citrate. The grids were examined using a Hitachi 7600 transmission electron microscope operating at 80 kV. Images were captured using an AMT 1 K×1 K CCD camera. Three to five sections from two rats per treatment were cut surrounding the injection sites and utilized for quantitative analysis.

### Bioluminescence Imaging

To study the effect of the microenvironment immediately after injury on the survival of grafted cells, a total of 7 rats underwent bioluminescence imaging. Three rats received hES-OPC injections after a 6.25 mm contusion injury, and 4 rats were injected with cells without injury, i.e. laminectomy only. For *in vivo* tracking of luciferase intensity, each animal was imaged with the Xenogen IVIS-200 Optical In Vivo Imaging System (Caliper Life Sciences, Hopkinton, MA). At each imaging session, rats received an i.p. injection of the ketamine mixture, as described above. At the onset of anesthesia, animals received another i.p. injection of D-luciferin (300 mg/kg) in normal physiological saline solution and were placed in the chamber of a charge-coupled device-camera system after a small skin incision to expose the region of interest. In the following 40 minutes, a series of luminescence images representing luciferin light intensity were acquired, and the images with peak intensities were analyzed. The Living Image 3D Analysis (Xenogen Corporation, Caliper Life Sciences) software was used for image processing. The regions of interest on the images were selected and quantified in terms of total flux (photons/second). Background values were chosen to calculate the net flux (photons/second). The peak value for each rat was chosen for analysis and was merged with a corresponding reference image for anatomical localization.

### Statistical Analysis

For the BLI analyses, statistical significance was calculated using an unpaired two-tailed Student’s t-test. For the electrophysiological analysis, each time point after injury was normalized to the average of the two baseline recordings for each individual rat. For amplitude and latency bar plots, the values derived for the left and right limb of each rat were averaged to yield one amplitude and one latency value per rat. This was performed in order to safeguard against the confounding limb factor if only the left or right side were considered. The group mean percentage of baseline was compared between the two experimental groups using a one-tailed unpaired t-test. For histological analysis three to five fields per rat were randomly selected and staining intensity a number quantified using Metamorph 7.7.7 software (Universal Imaging).

## Results

### Transplanted OPCs Promote Repair of the Sensory Pathways

**Figure 1:**
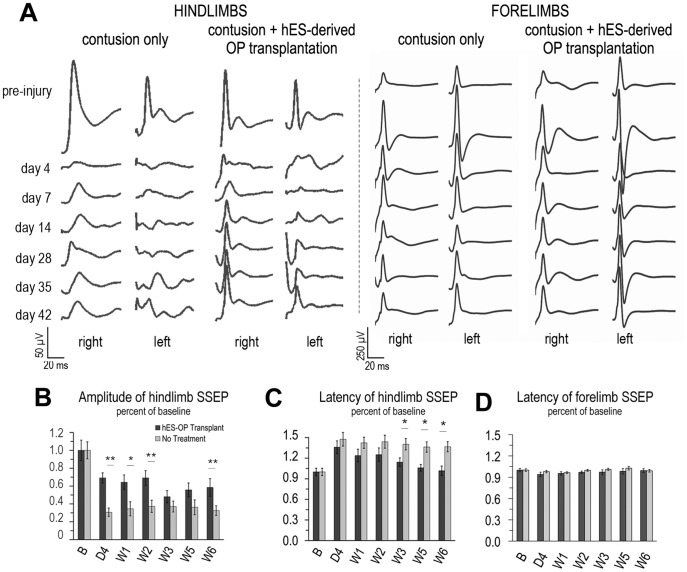
SSEP results showing functional improvements in spinal cord injured rats following transplantation of hES-derived OPCs compared with rats receiving no treatment. A) Representative mean SSEP sweeps from one rat from each experimental group. Left: SSEPs recorded upon hindlimb stimulation following T8 contusion; Right: internal controls due to forelimb stimulation. B) Amplitude (hindlimbs) results quantified for the two experimental groups showing a significant benefit for OPC transplantation versus no treatment. C) Latency (hindlimbs) results reveal that the OP-transplant group exhibited remyelination, as the time of the SSEP signal transduction reduces back to baseline values. This result is indicative of higher conductivity of axonal pathways, which leads to lower latencies for the OPC-transplant group. D) Latencies of forelimb SSEPs over time did not change post-injury in both groups indicating no harm to forelimb sensory pathways. *p<0.05 and **p<0.01.

SSEPs were used to evaluate the functional integrity of ascending sensory pathways following SCI and the transplantation of hES-derived OPCs. SSEPs are a quantitative way to assess the conduction of somatosensory pathways following trauma to the spinal cord. SSEPs were measured for each healthy rat prior to injury to acquire baseline recordings for the longitudinal study. Following SCI, the SSEPs of both the injury and transplantation group were significantly reduced in amplitude with increased latencies. These two parameters represent the amount of signal transduction through site of injury and the speed at which the signal travels, respectively. As a number of demyelinated but intact axons still exist following SCI, enhancements in SSEPs following OPC cell therapy would reveal a remyelination of spared axonal pathways that result in increased conductivity. **Figure 1** shows that the OPC-transplantation group exhibited an increase in amplitude over the course of 6 weeks post-injury that was accompanied by a reduction in latency that reached baseline values. The experimental group treated with OPCs exhibited less of an initial reduction in SSEP amplitude during the first 2 weeks of injury. At day 4 post-injury, the treated and untreated groups exhibited SSEP amplitudes 69±6% and 31±8% of baseline, respectively This difference persisted up to week 3 at which point no notable difference between groups was detected until week 6 (**Figure 1B**). By the sixth week post-transplant, the amplitude of SSEPs for the treatment group was significantly greater than the injury-only group (58±10% versus 33±5%, respectively; P = 0.009), which suggests a secondary period of recovery. As an internal control, we additionally recorded forelimb SSEPs to show that no damage was administered to forelimb pathways (**Figure 1B** **, left**).

The latency was also evaluated. This is the time from stimulation at the periphery to the appearance of the first positive peak of the SSEP waveform, and as such can be used as an indicator of the speed of conduction of sensory pathways. Here we show that the latencies of the control group increased as expected to 148±10% of baseline at day 4 and showed no improvement over the course of the 6 weeks studied (**Figure 1C**). However, the OPC-treatment group, which also exhibited an increased latency at day 4 (136±9% of baseline), showed a significant improvement with time, resulting in a latency by week 6 that was similar to the baseline prior to injury (102±6%). Since improvement occurred several weeks after the typical inflammatory-induced responses normally appear, these results suggest that the functional improvement of sensory pathways may be due to a more permanent recovery of the tissue. One possibility to explain the SSEP results is that hES OPC facilitate remyelination of spared axons by the transplanted hES-OPCs.

### Transplanted hES-OPCs Mature in the Spinal Cord

**Figure 2:**
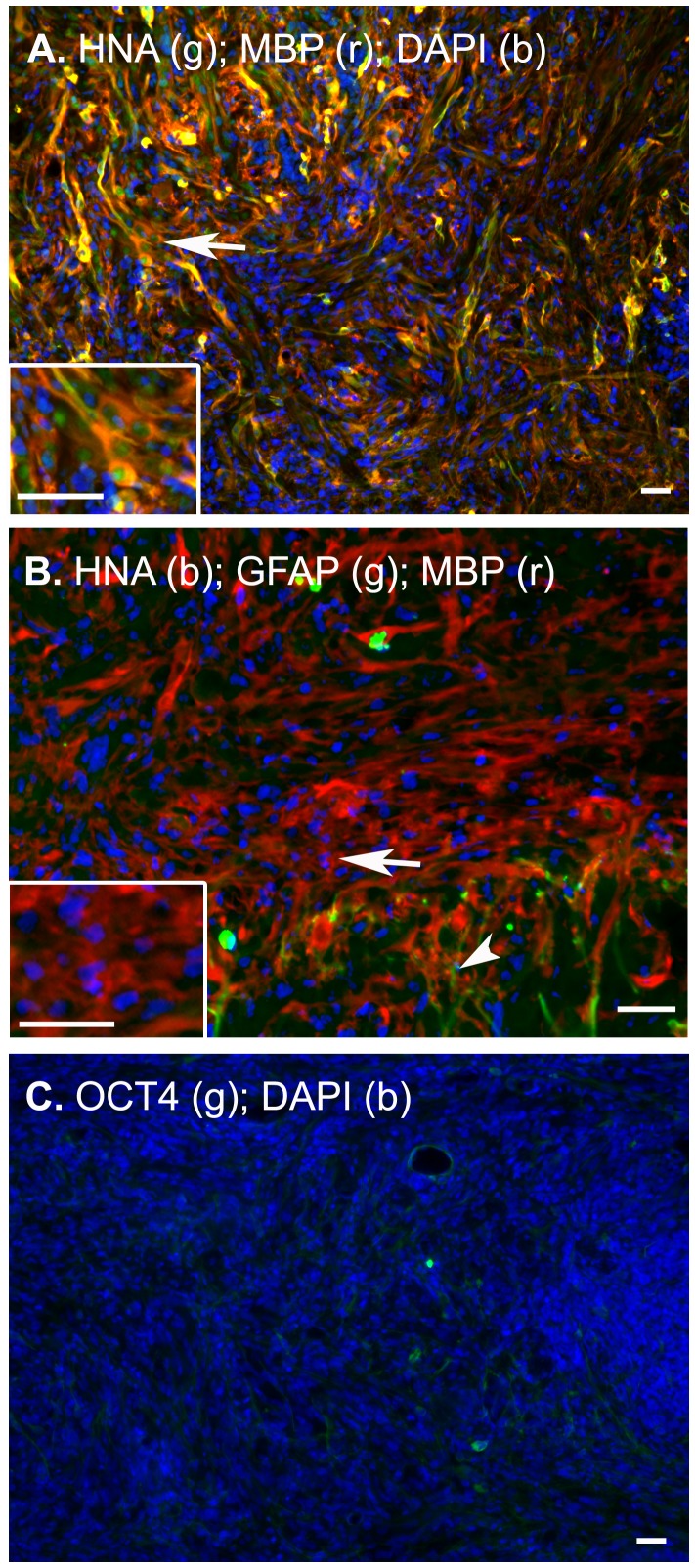
Immunohistochemical staining showing survival and cell type profiling of transplanted cells surrounding the injury epicenter approximately two months after injury. (A) Dual staining for human nuclear antigen (HNA, green) and myelin basic protein (MBP, red) verifies the survival of human OPCs in the rat tissue at the site of injection into the epicenter of the injury. Cells indicated by the arrow are shown at higher magnification in the insert. The majority of these cells are surrounded by intense MBP staining at the site of injection into the grey matter where MBP expression is normally low demonstrating the production of myelin in these areas. Nuclei of both the human and rat cells are identified by DAPI (blue). (B) Triple stains for HNA (blue), the astrocytic marker GFAP (green) and mature oligodendrocyte MBP (red) at these sights confirm that the majority of transplanted cells did not express the astrocyte marker GFAP. Instead astrocytic extensions (arrowhead) can be seen reaching into the area of injury from the surrounding white matter. Insert indicated by the arrow in B shows that human OPCs indicated by the blue HNA stain were primarily located at sites of intense MBP staining. (C) Staining for the pluripotent marker OCT4 (green) was not detected in the tissue.

To study the behavior of transplanted cells in these rats, immunohistochemical analyses were performed postmortem. OPC-treated animals demonstrated positive human nuclear antigen (**HNA**) expression, indicating survival of grafted OPCs at the end of these 6 week experiments (**Figure 2A**). The majority of the HNA^+^ cells were surrounded by MBP staining suggesting that OPCs differentiated into myelin-producing oligodendrocytes. The expression of MBP in the transplanted HNA^+^MBP^+^ double-labeled cells would be indicative of the myelin synthesis. Spinal cords transplanted with saline only or heat killed OPCs did not demonstrate HNA staining. Likewise, rats injected with human fibroblasts showed no indication of fibroblast survival past one week as demonstrated by the lack of HNA positive cells in these cords (data not shown).

Some studies have suggested that OPCs retain the ability to differentiate into glial [11]. To determine whether our transplanted cells retain this potential, we stained for the astrocyte marker, GFAP. Triple-immunostaining for HNA, GFAP, and MBP are shown in **Figure 2B**. The HNA positive cells did not stain for GFAP but did stain for MBP, indicating that the transplanted cells did not differentiate into astrocytes. To further rule out any possibility of reversion or undifferentiated stem cells in our transplants, we performed additional immunostains for OCT4, a marker of stem cell pluripotency. No OCT4 staining was observed (**Figure 2C**), which indicated that the majority of transplanted cells did not possess stem cell-like self-renewal properties but were differentiated along the oligodendrocyte lineage.

### Improved Anatomical Morphology in hES-derived OPC-treated Animals

**Figure 3:**
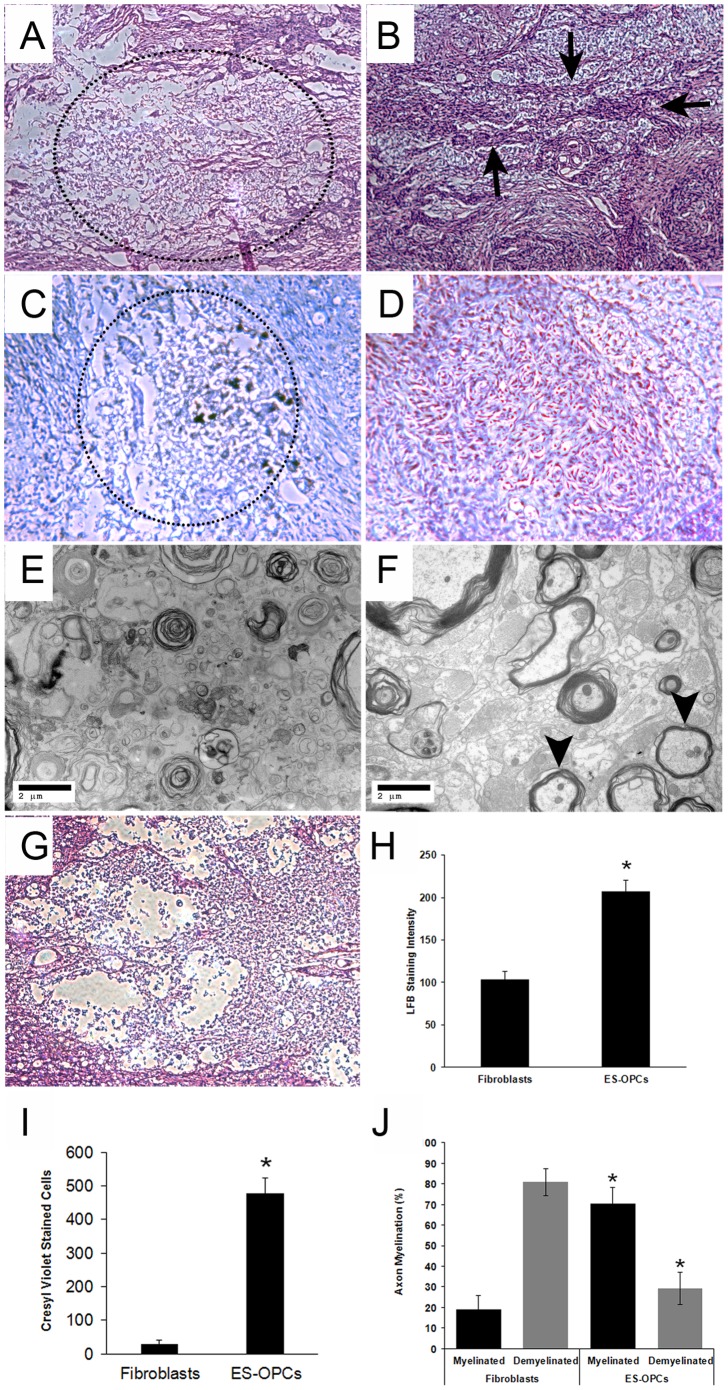
Light and electron microscopy. Sagittal sections corresponding to the center of injury approximately 2 months after injury. H&E stained paraffin sections of spinal cords from animals subjected to contusive SCI and treated with human fibroblasts (A) or hES-derived OPCs (B) Arrows indicate areas of neuron like tracts transversing the center of injury in OPC treated rats. This is consistent with LFB (blue) and cresyl violet (purple) staining which showed significantly more cavitation (dotted circles) in the human fibroblast-treated group (C) compared to the hES-derived OPC group (D). Specifically the fibroblast treated groups shows overall loss of tissue integrity with reduced LFB and cresyl violet staining, while the OPC treated group shows the presence of neurons, stained with cresyl violet surrounded by LFB staining. Transmission electron microscopy of sagittal sections of spinal cords also showed disrupted myelin for the fibroblast-treated group (E), whereas remyelination with thin, compact sheaths was observed for the hES-OPC group (F, arrowheads). Rats treated with heat-killed OPCs (G) were similar to fibroblast controls. Magnification: (A–D, G) 40x and (E, F) 5000x. These results were verified by quantitative analysis demonstrating (H) an increase in myelin staining by LFB, (I) the number of axons identified by cresyl violet and (J) by the extent of myelination demonstrated by electron microscopy in the hES-OPC group compared to controls. Error bars represent standard deviations. Asterisks denotes statistical significance between fibroblast and ES-OPC groups (P<0.00).

To study gross changes in anatomical recovery, we analyzed paraformaldehyde preserved spinal cords embedded in paraffin for optimal morphological preservation. For this purpose, standard H&E and LFB staining was performed on sagittal sections of the spinal cords excised after transplantation. Indeed, it has been speculated in certain circumstances that the tissue repair detected by stem cell-based transplants is nonspecific to the cells injected and instead induced by growth factors they provide the wound. As the recovery in SSEP showed both short-term as well as long-term recovery, live human fibroblasts were used as controls to test the cell specific interactions between the hES-derived OPCs and the host tissue in the following experiments. Results showed that at the epicenter of injury more cavitation was observed in the fibroblast-treated control group and heat-killed OPCs compared to the group treated with hES-derived OPCs (**Figure 3A–B, and G**). This was shown by the lack of human or rat cells in this region and by the overall loss of the integrity of the spinal cord in the fibroblast controls. Similar reductions in cavitation have been shown for implanted bone marrow mesenchymal stem cells in experimental SCI where there survival is poor [37]. In comparison, evidence suggests that some continuity of the neural circuitry can be detected through the epicenter in the sagittal sections of SCI treated with OPCs. This tissue was also examined with LFB, a dye that stains for the lipoprotein component of myelin and counterstained with cresyl violet, which stains the Nissl substance in neurons. The loss of blue stain at the epicenter in control spinal cords signified the loss of myelin to this area compared to those transplanted with OPCs (**Figure 3C–D**). In this case, OPC treated rats demonstrated higher levels of LFB staining than controls (**Figure 3H**). Moreover, unlike controls which were nearly devoid of any neural bodies, as shown by the absence of cresyl violet, OPC treated rats demonstrated an increased presence of the cells (**Figure 3I**). These results therefore confirm that the presence of human OPCs appear to facilitate some anatomical recovery in these rats and in a cell specific manner.

We verified new myelin formation after OPC transplantations using TEM (**Figure 3E–F**). New myelin that is generated after injury forms thinly packed sheaths around axons, whereas native myelin that survived the contusion is characterized by densely packed layers of myelin around axons. This difference in thickness around axons has been used as an indicator of myelination by graft cells [36]. Rats receiving the OPC injection exhibited new myelin formation that was characterized by thinly packed sheaths. Furthermore, these could be distinguished from the presence of thick, dense sheaths representing native myelin that survived the injury. In contrast, rats that received heat-killed OPCs showed neither surviving, thick myelin sheaths nor evidence of new myelin formation. Instead, they showed primarily disrupted or damaged myelin sheaths. Quantitative analysis confirmed more myelination in the ES-OPCs compared to the control group (**Figure 3J**). Thus, results from EM were consistent with light microscopy examination of these spinal cords. However, it remains to be determined whether the myelination in these experiments was generated from the human OPCs or endogenous tissue even though human fibroblasts or heat-killed OPCs were unable to facilitate these processes.

### Transplanted hES-OPCs Survive Transplantation in an Acute SCI Injury Model

**Figure 4:**
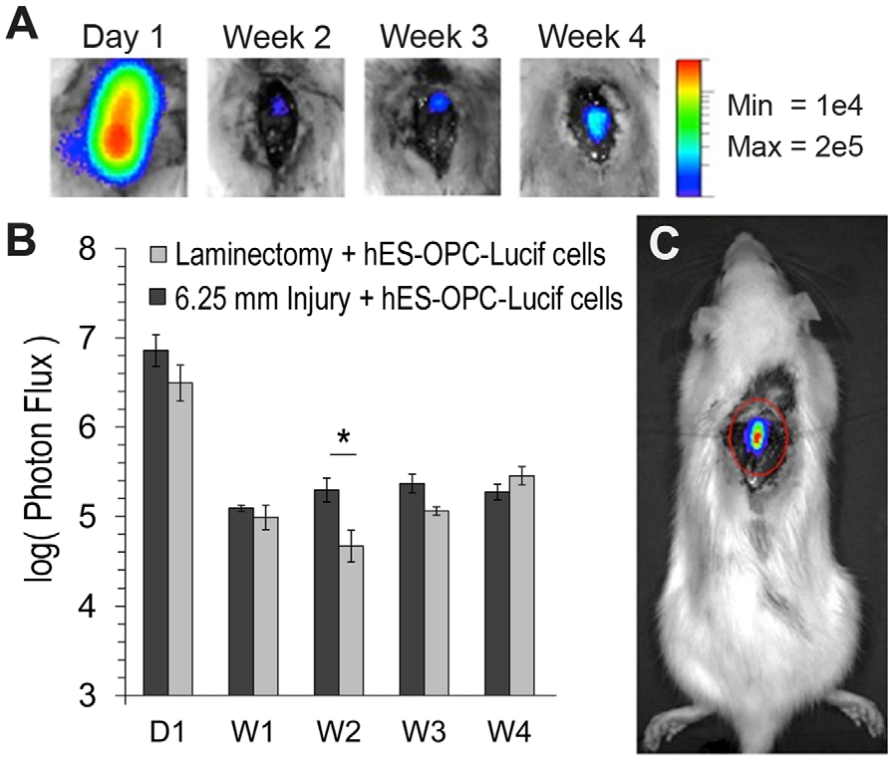
Bioluminescence of hES derived OPC-treated groups. **Bioluminescence was followed at six different time points over a period of 4 weeks.** A) Images taken at each time point that depict the bioluminescent activity of the transplanted cells. B) Total flux (photons/second) measured at various time points of the OPC-treated injury and laminectomy-only groups. Error bars represent standard deviations. C) An image of an animal showing bioluminescent activity at the site of OPC transplantation. Asterisk denotes a statistical significance in ES-OPC survival at two weeks between laminectomy and injury group (p<0.05).

To examine if hES cell-derived OPCs could survive in a recently injured (2 hours) spinal cord after transplantation, two groups of rats were studied: one group receiving only a laminectomy and another group receiving a mild contusive injury. OPCs were transplanted into both groups 2 hours after laminectomy or injury and were monitored using BLI weekly for 4 weeks. BLI allowed us to verify the survival and proliferation of the cells following transplantation. The first imaging session was performed 1 day after transplantation of the OPCs. Acquired images (**Figure 4**) at day-1 showed very intense luciferase activity, which was concentrated at the site of transplantation (7.22×10^6^±3.62×10^6^ photons/sec and 3.12×10^6^±1.84×10^6^ photons/sec for the laminectomy and injury groups, respectively). After two weeks, a significant difference in luminescence between the control and injury groups emerged. At this time, bioluminescence for the laminectomy group showed 2.7% of the intensity on the day 1 responses, whereas the luminescence of the injury group was 1.5% of the day 1 intensity. These results demonstrated that OPC survival was likely affected by inflammation and secondary injury mechanisms within the first week of trauma. However, by week 3 post-implantation, there was no significant difference between groups. The observed trends indicated that some cells survived the initial transplantation and secondary injury phase and then continued to proliferate *in vivo* for two weeks such that by week 4, there was no observable difference in cell survival between injury and non-injured groups.

## Discussion

In the present study, we showed that for a moderate contusive spinal cord injury, surviving cells that were transplanted 2 hours after injury were still detected after 4 weeks and that such acutely administered cell transplants may reduce the amount of secondary injury and partially restore the functionality of the ascending sensory pathways in the spinal cord. Takahashi *et al.* demonstrated that BLI technology can successfully detect a minimum of 1,000 mouse cells in a model of contused SCI [38]. Our results are similar to previous reports of stem cell-derived cell transplants in mouse models of SCI [17], [38] for which BLI intensity was high on the first day after transplantation but significantly decreased during the following two weeks. At 2 weeks post-transplantation, bioluminescence was significantly higher in the laminectomy sham group compared to the injured group; this may be due to inflammation and other secondary injury mechanisms that occur following spinal cord trauma that lead to a hostile microenvironment for the transplanted cells. However, there was no statistically significant difference in the observed BLI intensity between the injury and laminectomy groups at subsequent time points. This indicates that although cell survival at 2 weeks was reduced due to the SCI, cells nonetheless survived the secondary injury phase and were able to proliferate, reaching cell counts comparable to that which was found in a healthy sham control by week 3. Thus, contrary to conventional beliefs, the contusive injury microenvironment almost immediately after injury (2 hours) can still accept transplants without affecting long-term cell survival.

One study using iPS cell-derived neurospheres for transplant after contusive SCI has reported some evidence of MEP improvement after SCI [17]. No study to date has evaluated the effect of stem cell derived transplants on the somatosensory pathways in SCI using electrophysiology, despite the fact that the somatosensory pathways receive the primary impact of the majority of contusive SCIs in humans. Somatosensory evoked potentials or SSEPs, are used to assess integrity of sensory tracts of the spinal cord. The improvement in the function of the sensory pathways can be seen by the increase in SSEP amplitude over the course of 6 weeks post-injury. Our results show for the first time the extent to which SSEP amplitudes were reduced in rats immediately following injury was less in rats receiving hES cell transplants compared to controls. This benefit in OPC treated rats is unlikely to be due to remyelination, as two weeks is not a sufficient time for immature OPCs to differentiate into mature OLs and produce sufficient and integrated myelin. However, others have shown the ability of progenitor cells to reduce acute inflammatory responses in damaged regions of the central nervous system (**CNS**). These studies have included the transplantation of neural precursor cells and neural-induced mesenchymal stem cells (**MSCs**) into the injured CNS, which result in reduced inflammation and fibrosis associated with secondary injury processes and improved neuronal regeneration [39], [40]. Although, the precise mechanisms through which these transplanted progenitors attenuate inflammation after SCI are still unclear, it is reasonable to infer that hES-derived OPCs may also possess some of the same anti-inflammatory properties that have been observed for other less differentiated progenitors.

Consistent with the changes in SSEP amplitude, the latencies of both groups were affected by their treatment. Initially, the SSEP latencies of both groups increased by approximately 40% at day 4 post-injury. This increase reflects the decrease in conductivity that results after injury due to damage to both the axons and their surrounding insulating myelin. Our results showed that following OPC transplantation, the latency of the OPC treatment group decreased to normal baseline values by week 6. Therefore, by 6 weeks after injury, the presence of transplanted OPCs may facilitate the remyelination of intact axons and restore their conductivity. This would be suggested by the increased conductivity via remyelination of spared axons that would then result in a decrease in the latencies of SSEPs, which are measured at the cortex due to stimulation rostral to the site of injury. Although SSEP latency recovered in OPC treated rats, SSEP amplitudes in these animals remained below baseline values. This is consistent with the persistence of the injury-induced cavity, which remains in treated animals such that any severed axons are unable to re-grow across the lesion.

In addition, it can be noted that the SSEPs of each animal did not always recover in a symmetrical fashion (**Figure 1A**). Although the contusion injury is induced at the midline, the natural progression of injury in all directions (laterally, proximodistally, or ventrodorsally) over time is unpredictable both in human and experimental models. The SSEP is an objective and sensitive tool to assess the integrity of the sensory pathways, which extends from periphery to the somatosensory cortex. It reflects the true electrical conductivity of the dorsal ascending pathways, which in turn reveals the progress of injury and not just the damage induced at the site of first impact. Therefore, it is reasonable to observe that a midline contusion in the spinal cord, which evolves into an asymmetrical injury over time, results in dissimilar SSEP recovery of the left and right sides.

It has been postulated that maximal amplitude improvement in SSEP is a function of the absolute number of axons that remain intact and that cavitation prevents the endogenous regrowth of axons across the lesion site [28]. Thus, the recovery in SSEP latency in OPC treated rats may indicate that this fraction of axons, which were non-functional due to demyelination, were successfully remyelinated and their conductivity was restored to pre-injury levels. The general somatic afferents transduce signals of touch, pressure, movement and body position from the periphery to the cortex. They are two main sensory pathways: one from the lower trunk and legs called fasciculus gracilis located in the medial dorsal column and one from the upper trunk and arms called fasciculus cuneatus located in the lateral dorsal column. The neurons (first-order) of the fasciculus gracilis and fasciculus cuneatus ascend uninterrupted in the dorsal column and terminate in their respective nuclei in the medulla, the nucleus gracilis and the nucleus cuneatus. Here, second-order neurons of the internal arcuate fibers originate and cross to the contralateral brainstem in the medulla, eventually synapsing in the thalamus and activating third-order neurons that reach the cortex. The latency we reported herein reflects only changes in first-order neurons of the spinal cord. We report the latency recovery of the injured hindlimbs after transplantation with ES-derived OPCs. To verify that this recovery is due to repair at the injury site, we underscore that the latency of the forelimb SSEPs remains unchanged (**Figure 1D**). This indicates that the function of second- and third-order neurons did not change over time for either experimental group. Therefore, the latency recovery of the OP-treated group can only be due to recovery of the first-order hindlimb neurons of the fascilus gracilis. In addition, because the untreated control group did not exhibit improved latency, we can conclude that the reduction in latency is due to remyelination of neurons, allowing faster conduction through the site of injury.

This is consistent with the histological analysis performed on spinal cords from euthanized animals at the end of the longitudinal BLI and SSEP studies. The tumorigenicity of hES-derived cell lineages has been a point of concern over the past decade and has been observed in various studies [41]. Our BLI results show that the transplanted OPCs did not migrate more than 1–2 mm from the locus of the injury or to other regions of the CNS, such as the brain. The pluripotent marker OCT4 was not detected in spinal cords treated with transplanted cells. Although this study incorporated a time in which tumor formation from ES cell transplantation can easily be detected it does not rule out the possibility of tumor formation had the study proceeded for a longer period of time. Thus, long-term experiments are required for this determination, which were not the intent of this study.

We also performed a series of immunostains for markers of oligodendrocytes, and myelin on slices of extracted spinal cord segments to verify the nature of our transplanted cells. ES-OPCs began to express MBP two weeks after transplantation. This suggests the presence of mature human OLs derived from the transplanted OPCs. Others have reported that stem cell-derived OPCs differentiate into other neural subtypes including astrocytes [11]. However, the human transplanted OPCs identified by human specific antigen did not show the presence of GFAP^+^ cells, indicating that the transplanted OPCs did not give rise to astrocytes.

The injured spinal cord is known to express a host of factors that inhibit the outgrowth and differentiation of OLs at the site of injury. To maximize the remyelination ability of our transplanted OPCs, additional steps can include a combination of cell therapy with the manipulation of growth factors or various signaling molecules. For example, bone morphogenic proteins (BMPs) expressed by reactive astrocytes inhibit the differentiation of OPCs to OLs while promoting the formation of astrocytes [42], and the release of BMP is drastically increased following SCI. Blocking BMP activity by the antagonist noggin reversed the effect and promoted OPCs to follow the OL lineage [43]. Reactive astrocytes in the glial scar may also inhibit OPCs via the release of tumor necrosis factor-α [44].

Finally, we were able to observe the formation of new thinly wrapped myelin sheaths around axons for an OPC treated rat using TEM. Because no reliable myelin associated antibodies that can distinguish human and rat exist for histological purposes, it is not possible to distinguish myelin that is produced by the injected OPCs from endogenously produced myelin. However, it is promising that newly formed myelin was only observed in the rat that received the OPC injection. Furthermore, because our immunohistochemical evaluation verified that our injected cells did form myelin-producing OLs, we can infer that at least a portion of these OLs functioned to remyelinate axons.

### Conclusion

There remains much to be investigated regarding how stem cells mediate recovery in spinal cord injured patients. The ability to track cell survival and migration serves to augment current knowledge about the mechanisms of stem cell therapies. Here, we have shown for the first time improvements in functional electrophysiological behavior in conjunction with biological evidence of both OL differentiation and myelin formation *in vivo*. Combining this evidence with additional studies in order to elucidate the full mechanisms of stem cell integration and spinal cord repair will inevitably lead to un-matched treatment for patients with SCI in the near future.

## References

[1] Sahni V, Kessler JA (2010) Stem cell therapies for spinal cord injury. Nature Reviews Neurology 6: 363–372. 20551948 10.1038/nrneurol.2010.73 PMC3755897

[2] Tsuji O, Miura K, Fujiyoshi K, Momoshima S, Nakamura M, et al (2011) Cell Therapy for Spinal Cord Injury by Neural Stem/Progenitor Cells Derived from iPS/ES Cells. NeuroTherapeutics 8: 668–676. 21909829 10.1007/s13311-011-0063-z PMC3250290

[3] Hwang D, Kim B, Kim E, Lee S, Joo I, et al (2009) Transplantation of human neural stem cells transduced with Olig2 transcription factor improves locomotor recovery and enhances myelination in the white matter of rat spinal cord following contusive injury. BMC neuroscience 10: 117. 19772605 10.1186/1471-2202-10-117 PMC2758886

[4] Ogawa D, Okada Y, Nakamura M, Kanemura Y, Okano HJ, et al (2009) Evaluation of human fetal neural stem/progenitor cells as a source for cell replacement therapy for neurological disorders: Properties and tumorigenicity after long term in vitro maintenance. Journal of Neuroscience Research 87: 307–317. 18972448 10.1002/jnr.21843

[5] Abematsu M, Tsujimura K, Yamano M, Saito M, Kohno K, et al (2010) Neurons derived from transplanted neural stem cells restore disrupted neuronal circuitry in a mouse model of spinal cord injury. The Journal of clinical investigation 120: 3255. 20714104 10.1172/JCI42957 PMC2929730

[6] Kumagai G, Okada Y, Yamane J, Nagoshi N, Kitamura K, et al (2009) Roles of ES cell-derived gliogenic neural stem/progenitor cells in functional recovery after spinal cord injury. PLoS One 4: e7706. 19893739 10.1371/journal.pone.0007706 PMC2768792

[7] Rossi SL, Nistor G, Wyatt T, Yin HZ, Poole AJ, et al (2010) Histological and functional benefit following transplantation of motor neuron progenitors to the injured rat spinal cord. PLoS One 5: e11852. 20686613 10.1371/journal.pone.0011852 PMC2912300

[8] Geron (2010) Safety Study of GRNOPC1 in Spinal Cord Injury. In: NIH, editor. USA.

[9] Letzen BS, Liu C, Thakor NV, Gearhart JD, All AH, et al (2010) MicroRNA expression profiling of oligodendrocyte differentiation from human embryonic stem cells. PloS one 5: e10480. 20463920 10.1371/journal.pone.0010480 PMC2864763

[10] Kerr CL, Letzen BS, Hill CM, Agrawal G, Thakor NV, et al (2010) Efficient differentiation of human embryonic stem cells into oligodendrocyte progenitors for application in a rat contusion model of spinal cord injury. International Journal of Neuroscience 120: 305–313. 20374080 10.3109/00207450903585290

[11] Erceg S, Ronaghi M, Oria M, García Roselló M, Aragó MAP, et al (2010) Transplanted oligodendrocytes and motoneuron progenitors generated from human embryonic stem cells promote locomotor recovery after spinal cord transection. Stem Cells 28: 1541–1549. 20665739 10.1002/stem.489 PMC2996083

[12] Sharp J, Frame J, Siegenthaler M, Nistor G, Keirstead HS (2010) Human Embryonic Stem Cell Derived Oligodendrocyte Progenitor Cell Transplants Improve Recovery after Cervical Spinal Cord Injury. Stem Cells 28: 152–163. 19877167 10.1002/stem.245 PMC3445430

[13] Keirstead HS, Nistor G, Bernal G, Totoiu M, Cloutier F, et al (2005) Human embryonic stem cell-derived oligodendrocyte progenitor cell transplants remyelinate and restore locomotion after spinal cord injury. The Journal of Neuroscience 25: 4694. 15888645 10.1523/JNEUROSCI.0311-05.2005 PMC6724772

[14] Faulkner J, Keirstead HS (2005) Human embryonic stem cell-derived oligodendrocyte progenitors for the treatment of spinal cord injury. Transplant Immunology 15: 131–142. 16412957 10.1016/j.trim.2005.09.007

[15] Okada S, Ishii K, Yamane J, Iwanami A, Ikegami T, et al (2005) In vivo imaging of engrafted neural stem cells: its application in evaluating the optimal timing of transplantation for spinal cord injury. The FASEB journal 19: 1839. 16141363 10.1096/fj.05-4082fje

[16] Moloney TC, Dockery P, Windebank AJ, Barry FP, Howard L, et al (2010) Survival and immunogenicity of mesenchymal stem cells from the green fluorescent protein transgenic rat in the adult rat brain. Neurorehabilitation and Neural Repair 24: 645. 20378924 10.1177/1545968309357745

[17] Nori S, Okada Y, Yasuda A, Tsuji O, Takahashi Y, et al (2011) Grafted human-induced pluripotent stem-cell–derived neurospheres promote motor functional recovery after spinal cord injury in mice. Proceedings of the National Academy of Sciences 108: 16825–16830. 10.1073/pnas.1108077108 PMC3189018 21949375

[18] Curt A, Keck ME, Dietz V (1998) Functional outcome following spinal cord injury: significance of motor-evoked potentials and ASIA scores. Archives of physical medicine and rehabilitation 79: 81–86. 9440423 10.1016/s0003-9993(98)90213-1

[19] Dietz V, Wirz M, Colombo G, Curt A (1998) Locomotor capacity and recovery of spinal cord function in paraplegic patients: a clinical and electrophysiological evaluation. Electroencephalography and Clinical Neurophysiology/Electromyography and Motor Control 109: 140–153. 9741805 10.1016/s0924-980x(98)00002-2

[20] Michelini E, Cevenini L, Mezzanotte L, Roda A (2009) Luminescent probes and visualization of bioluminescence. Methods in Molecular Biology 574: 1–13. 19685295 10.1007/978-1-60327-321-3_1

[21] Sher F, van Dam G, Boddeke E, Copray S (2009) Bioluminescence Imaging of Olig2 Neural Stem Cells Reveals Improved Engraftment in a Demyelination Mouse Model. Stem Cells 27: 1582–1591. 19544465 10.1002/stem.76

[22] Dothager RS, Flentie K, Moss B, Pan MH, Kesarwala A, et al (2009) Advances in bioluminescence imaging of live animal models. Current opinion in biotechnology 20: 45–53. 19233638 10.1016/j.copbio.2009.01.007 PMC2680462

[23] Bottenstein JE, Sato GH (1979) Growth of a rat neuroblastoma cell line in serum-free supplemented medium. Proceedings of the National Academy of Sciences 76: 514. 10.1073/pnas.76.1.514 PMC382972 284369

[24] Amit M, Margulets V, Segev H, Shariki K, Laevsky I, et al (2003) Human feeder layers for human embryonic stem cells. Biology of reproduction 68: 2150–2156. 12606388 10.1095/biolreprod.102.012583

[25] Hovatta O, Mikkola M, Gertow K, Strömberg AM, Inzunza J, et al (2003) A culture system using human foreskin fibroblasts as feeder cells allows production of human embryonic stem cells. Human Reproduction 18: 1404–1409. 12832363 10.1093/humrep/deg290

[26] Agrawal G, Iyer S, All AH (2009) A comparative study of recording procedures for motor evoked potential signals. 31st Annual International Conference of the IEEE EMBS. Minneapolis, Minnesota: IEEE. 2086–2089. 10.1109/IEMBS.2009.5333953 19964577

[27] Agrawal G, Kerr C, Thakor NV, All AH (2010) Characterization of graded multicenter animal spinal cord injury study contusion spinal cord injury using somatosensory-evoked potentials. Spine 35: 1122. 20354478 10.1097/BRS.0b013e3181be5fa7 PMC2871968

[28] Agrawal G, Sherman D, Maybhate A, Gorelik M, Kerr DA, et al (2010) Slope analysis of somatosensory evoked potentials in spinal cord injury for detecting contusion injury and focal demyelination. Journal of Clinical Neuroscience 17: 1159–1164. 20538464 10.1016/j.jocn.2010.02.005

[29] Agrawal G, Thakor NV, All AH (2009) Evoked potential versus behavior to detect minor insult to the spinal cord in a rat model. Journal of Clinical Neuroscience 16: 1052–1055. 19419872 10.1016/j.jocn.2008.08.009

[30] All AH, Agrawal G, Walczak P, Maybhate A, Bulte JWM, et al (2010) Evoked potential and behavioral outcomes for experimental autoimmune encephalomyelitis in Lewis rats. Neurological sciences 31: 595–601. 20508959 10.1007/s10072-010-0329-y PMC3036170

[31] All AH, Walczak P, Agrawal G, Gorelik M, Lee C, et al (2009) Effect of MOG sensitization on somatosensory evoked potential in Lewis rats. Journal of the neurological sciences 284: 81–89. 19423134 10.1016/j.jns.2009.04.025 PMC2721914

[32] Bazley FA, All AH, Thakor NV, Kerr C, Maybhate A (2011) Plasticity Associated Changes in Cortical Somatosensory Evoked Potentials following Spinal Cord Injury in Rats. 33rd Annual International Conference of the IEEE EMBS. Boston, Massachusetts: IEEE. 2005–2008. 10.1109/IEMBS.2011.6090564 22254728

[33] Bazley FA, Hu C, Maybhate A, Pourmorteza A, Pashai N, et al. (2012) Electrophysiologal evaluation of sensory and motor pathways after incomplete unilateral spinal cord contusion. Journal of Neurosurgery: Spine In press. 10.3171/2012.1.SPINE11684 22303873

[34] Maybhate A, Hu C, Bazley FA, Yu Q, Thakor NV, et al. (2011) Potential long-term benefits of acute hypothermia after spinal cord injury: Assessments with somatosensory-evoked potentials. Critical Care Medicine 39. 10.1097/CCM.0b013e318232d97e PMC3261348 22001581

[35] Walczak P, All AH, Rumpal N, Gorelik M, Kim H, et al (2011) Human glial restricted progenitors survive, proliferate, and preserve electrophysiological function in rats with focal inflammatory spinal cord demyelination. Glia 59: 499–510. 21264955 10.1002/glia.21119 PMC3079958

[36] Keirstead H, Nistor G, Bernal G, Totoiu M, Cloutier F, et al (2005) Human embryonic stem cell-derived oligodendrocyte progenitor cell transplants remyelinate and restore locomotion after spinal cord injury. Journal of Neuroscience 25: 4694. 15888645 10.1523/JNEUROSCI.0311-05.2005 PMC6724772

[37] Zeng X, Zeng Y, Ma Y, Lu L, Du B, et al (2011) Bone Marrow Mesenchymal Stem Cells in a Three-Dimensional Gelatin Sponge Scaffold Attenuate Inflammation, Promote Angiogenesis, and Reduce Cavity Formation in Experimental Spinal Cord Injury. Cell Transplantation, 20 11: 1881–1899. 10.3727/096368911X566181 21396163

[38] Takahashi Y, Tsuji O, Kumagai G, Hara CM, Okano HJ, et al (2011) Comparative study of methods for administering neural stem/progenitor cells to treat spinal cord injury in mice. Cell transplantation 20: 727–739. 21054930 10.3727/096368910X536554

[39] Einstein O, Grigoriadis N, Mizrachi-Kol R, Reinhartz E, Polyzoidou E, et al (2006) Transplanted neural precursor cells reduce brain inflammation to attenuate chronic experimental autoimmune encephalomyelitis. Experimental neurology 198: 275–284. 16472805 10.1016/j.expneurol.2005.11.007

[40] Park S, Lee Y, Lee S, Lee D, Choi K, et al. (2012) Functional recovery after spinal cord injury in dogs treated with a combination of Matrigel and neural-induced adipose-derived mesenchymal Stem cells. Cytotherapy. 10.3109/14653249.2012.658913 22348702

[41] Arnhold S, Klein H, Semkova I, Addicks K, Schraermeyer U (2004) Neurally selected embryonic stem cells induce tumor formation after long-term survival following engraftment into the subretinal space. Investigative ophthalmology & visual science 45: 4251–4255. 15557428 10.1167/iovs.03-1108

[42] Lü HZ, Wang YX, Zou J, Li Y, Fu SL, et al (2010) Differentiation of neural precursor cell-derived oligodendrocyte progenitor cells following transplantation into normal and injured spinal cords. Differentiation 80: 228–240. 20850923 10.1016/j.diff.2010.09.179

[43] Wang Y, Cheng X, He Q, Zheng Y, Kim DH, et al (2011) Astrocytes from the Contused Spinal Cord Inhibit Oligodendrocyte Differentiation of Adult Oligodendrocyte Precursor Cells by Increasing the Expression of Bone Morphogenetic Proteins. The Journal of Neuroscience 31: 6053. 21508230 10.1523/JNEUROSCI.5524-09.2011 PMC3081104

[44] Su Z, Yuan Y, Chen J, Zhu Y, Qiu Y, et al (2011) Reactive astrocytes inhibit the survival and differentiation of oligodendrocyte precursor cells by secreted TNF-α. Journal of Neurotrauma 28: 1089–1100. 21309692 10.1089/neu.2010.1597

